# Local progress towards achieving the End TB targets in Ethiopia: a geospatial analysis

**DOI:** 10.1093/ije/dyaf157

**Published:** 2025-09-04

**Authors:** Haileab Fekadu Wolde, Archie C A Clements, Kefyalew Addis Alene

**Affiliations:** School of Population Health, Faculty of Health Sciences, Curtin University, Bentley, Western Australia, Australia; Geospatial and Tuberculosis Research Team, The Kids Research Institute, Nedlands, Western Australia, Australia; Queen’s University Belfast, Belfast, United Kingdom; School of Population Health, Faculty of Health Sciences, Curtin University, Bentley, Western Australia, Australia; Geospatial and Tuberculosis Research Team, The Kids Research Institute, Nedlands, Western Australia, Australia

**Keywords:** End TB, spatial analysis, Ethiopia

## Abstract

**Background:**

Country-level estimates can mask local geographic variations in progress toward achieving World Health Organization’s End TB targets. This study aimed to identify spatial variations in progress toward achieving the TB incidence reduction target at a district level in Ethiopia.

**Methods:**

A Bayesian linear regression model with a conditional autoregressive prior structure was developed to identify drivers of spatial variations in TB incidence reduction across districts and to identify spatial patterns and variations in TB incidence reduction across Ethiopia from 2015 to 2020.

**Results:**

The national average TB incidence reduction was 31%. Ten out of 14 regions achieved a reduction of >20% in TB incidence. Out of 641 districts, 395 (61.6%) met the 20% reduction target, predominantly in the Oromia, Amhara, and South Ethiopia regions. Spatial clustering of decreased incidence reduction was noted in the Afar, Benishangul-Gumuz, and Somali regions. Factors associated with the percentage reduction in TB incidence include a 1% increase in the proportion of individuals with good TB knowledge [β: 4.23%; 95% credible interval (CrI): 1.6, 6.9], a 1-unit increase in the TB service readiness index (β: 3.41%; 95% CrI: 0.89, 6.1), and a 1-km increase in the distance from the international border (β: 2.63%; 95% CrI: 0.02, 5.10).

**Conclusion:**

Geographic disparities in TB incidence reduction persist in Ethiopia, with only some districts achieving the national reduction targets. Targeted interventions, such as improving TB service readiness and enhancing awareness through education, are crucial to addressing these gaps, particularly in regions such as Afar, Benishangul-Gumuz, and Somali.

Key Messages

- We examined how the reduction in tuberculosis (TB) incidence varied across districts in Ethiopia.
- Several districts in Ethiopia, particularly those in border regions, showed limited progress in reducing TB incidence.
- Targeted interventions and improved TB services and awareness are needed to address local gaps in TB incidence reduction in Ethiopia.

## Background

Tuberculosis (TB) is the leading cause of death from a single infectious agent worldwide [1]. According to the recent World Health Organization (WHO) report, Ethiopia is one of the world’s 30 high-TB- and TB/HIV-burden countries [2]. The TB incidence was reported to be 119 per 100 000 population in 2022 and a total of 19 000 people died due to TB in the same year in Ethiopia [1]. Different subnational surveys in the country have found the burden of TB ranging from 78 to 311 cases per 100 000 population [3–11].

As part of a continuous effort to end TB, all Member States of the WHO and the United Nations (UN) committed in 2014 and 2015 to ending the TB epidemic through their adoption of the WHO’s End TB Strategy and the UN Sustainable Development Goals [12]. The first End TB Strategy milestones for reductions in the TB disease burden were a 35% reduction in the total number of deaths caused by TB and a 20% reduction in the TB incidence rate by 2020 compared with levels in 2015 [13]. These targets have not yet been reached, either globally or in most of the WHO regions and countries [14, 15]. Nevertheless, Ethiopia is among a few countries with a high TB burden that has demonstrated a consistent decline in TB incidence. The incidence declined from 192 to 132 per 100 000 people between 2015 and 2020, which is a 5% average annual decline [16]. This makes Ethiopia one of the seven high-TB-burden countries that achieved the WHO’s 2020 End TB Strategy milestone, with a 31.3% total reduction in incidence [15]. However, with this current rate of decline, ending the TB epidemic (<10 TB cases per 100 000 population) and reaching the 2035 targets may not be possible [17].

Progress towards achieving the End TB targets might vary sub-nationally, reflecting the geographical variation in TB incidence and mortality [18–23] and healthcare access [24, 25]. An understanding of the subnational variation could help in designing and applying targeted approaches that combine diagnostic, treatment, and preventive interventions in areas with poor progress toward achieving the programmatic targets. In the case of Ethiopia, a district-level analysis would be most relevant because districts are the administrative level at which local resources are allocated for each sector. Therefore, this study aimed to quantify district-level progress in the TB incidence reduction in Ethiopia between 2015 and 2020 by using a geospatial analytic approach.

## Methods

### Study setting

The study was conducted in Ethiopia, the second-most populous country in Africa, with a total population of >126 million [26]. The country has a tiered administrative system consisting of regional states (first level), zones (second level), districts (third level), and villages (fourth level). Ethiopia adopted the directly observed therapy, short-course strategy in 1997 following a successful pilot programme and the development of the first combined Tuberculosis and Leprosy Prevention and Control Program (NTLCP) manual. Ethiopia has endorsed the new post-2015 Global End TB Strategy and aligned its National TB Strategic Plan with the National Health Sector Transformation Plan. The National End TB Strategy aims to end the TB epidemic by reducing incident TB cases by 90% between 2015 and 2035 [27].

### Data sources

District-level TB data were obtained from the Health Management Information System (HMIS) managed by the NTLCP. TB cases are reported by district health offices to the Federal Ministry of Health on a quarterly basis through the HMIS. For this study, we analysed the aggregated count of all types of TB, including bacteriologically confirmed and clinically diagnosed new pulmonary TB, new extrapulmonary TB, multidrug-resistant TB, and relapse TB cases for the years 2015 and 2020. We used the change in the number of notified TB cases as a proxy for TB incidence reduction between 2015 and 2020. We calculated the percentage reduction in TB incidence by subtracting the 2020 value from the 2015 baseline value and dividing the difference by the baseline value. A district with a ≥20% incidence reduction in 2020 compared with 2015 was classified as having achieved the first milestone of the End TB target [12, 13].

#### Covariates

Potential covariates with country-wide representative data and plausibly associated with TB incidence reduction were obtained from satellite images and publicly available sources (Supplementary Table S1). Climatic variables, including temperature, precipitation, and wind speed, were acquired from the WorldClim website [28]. Additionally, data on access to healthcare facilities were sourced from the Malaria Atlas Project [29] and population density, estimated as the number of people per grid, was obtained from WorldPop [30]. Socio-demographic variables such as knowledge and attitude towards TB, wealth status, mass media exposure, educational status, alcohol consumption, kat chewing, cigarette smoking, and number of household members were obtained from 2011 and 2016 Ethiopian Demographic Health Surveys (EDHS; note, no accurate census data are available at a district level, necessitating the use of estimates from the EDHS) [31]. Because EDHS data are collected at an individual level, Bayesian Kriging interpolation was used to produce pixel-level raster data for the entire country, which were then aggregated at the district level. The distance to international borders, measured as the geodesic distance to the nearest international border in kilometres from the centroid of the districts, was obtained from the Demographic and Health Survey (DHS) spatial repository [32]. The Ethiopia Public Health Institute (EPHI) Stepwise approach to Surveillance (STEPS) survey was also used to measure the prevalence of selected non-communicable diseases such as hypertension, diabetes mellitus, and smoking. Indicators of the preparedness and readiness of health facilities such as readiness for chronic respiratory disease and TB were measured by using data from the EPHI Service Availability and Readiness Assessment survey [33].

### Geoprocessing and hotspot analysis

The data were georeferenced by using a geographical information system: ArcGIS V.10.8.2 software (ESRI, Redlands, California, USA). The Ethiopian Polygon Shapefile, which contains the defined polygons representing administrative boundaries of districts within Ethiopia, was used for geocoding. The geocoded data were linked to covariates. Spatial clustering of undetected TB cases was explored at a global scale by using Moran’s I statistic and at a local scale by using the Getis-Ord GI* statistic. These analyses were conducted by using tools provided in ArcGIS Pro.

### Spatial model

A Bayesian spatial linear regression model was constructed to analyse the percentage reduction in TB incidence across districts. The model was defined as follows:

$$Y_{i}=a+\beta X_{i}+U_{i}+V_{i}$$

where *Y_i_* represents the percentage reduction in TB incidence for district *i*, *α* is the intercept, *βX_i_* denotes the fixed effects of covariates, and *U_i_* and *V_i_* represent the random effects. The spatially structured random-effects *U_i_* captures potential spatial autocorrelation, assuming that neighbouring districts tend to exhibit similar reductions in TB incidence. This spatial dependence was modelled by using a conditional autoregressive structure [34], with the neighbourhood matrix defined by common district boundaries. The unstructured random-effect *V_i_* accounts for uncorrelated noise.

Priors were assigned to all parameters in the model due to the Bayesian nature of the analysis. A non-informative uniform prior was used for the intercept α and normal priors with a mean of 0 and a precision (inverse of variance) = 1 × 10^−4^ were used for each $\beta_{j}$ coefficient. The precision parameters for the spatially structured and unstructured random effects were given non-informative gamma distributions with shape and scale parameters set at 0.001. Parameter estimation was conducted by using the Integrated Nested Laplace Approximation (INLA) approach in R (R-INLA). Various model specifications were tested and the best-fitting model was selected based on the Deviance Information Criterion (DIC), with the model having the lowest DIC being chosen for its superior fit and parsimony. All analyses were carried out by using R and ArcGIS software. Full modelling details can be found in Supplementary Material 2.

## Results

### Regional-level progress

**Figure 1.**
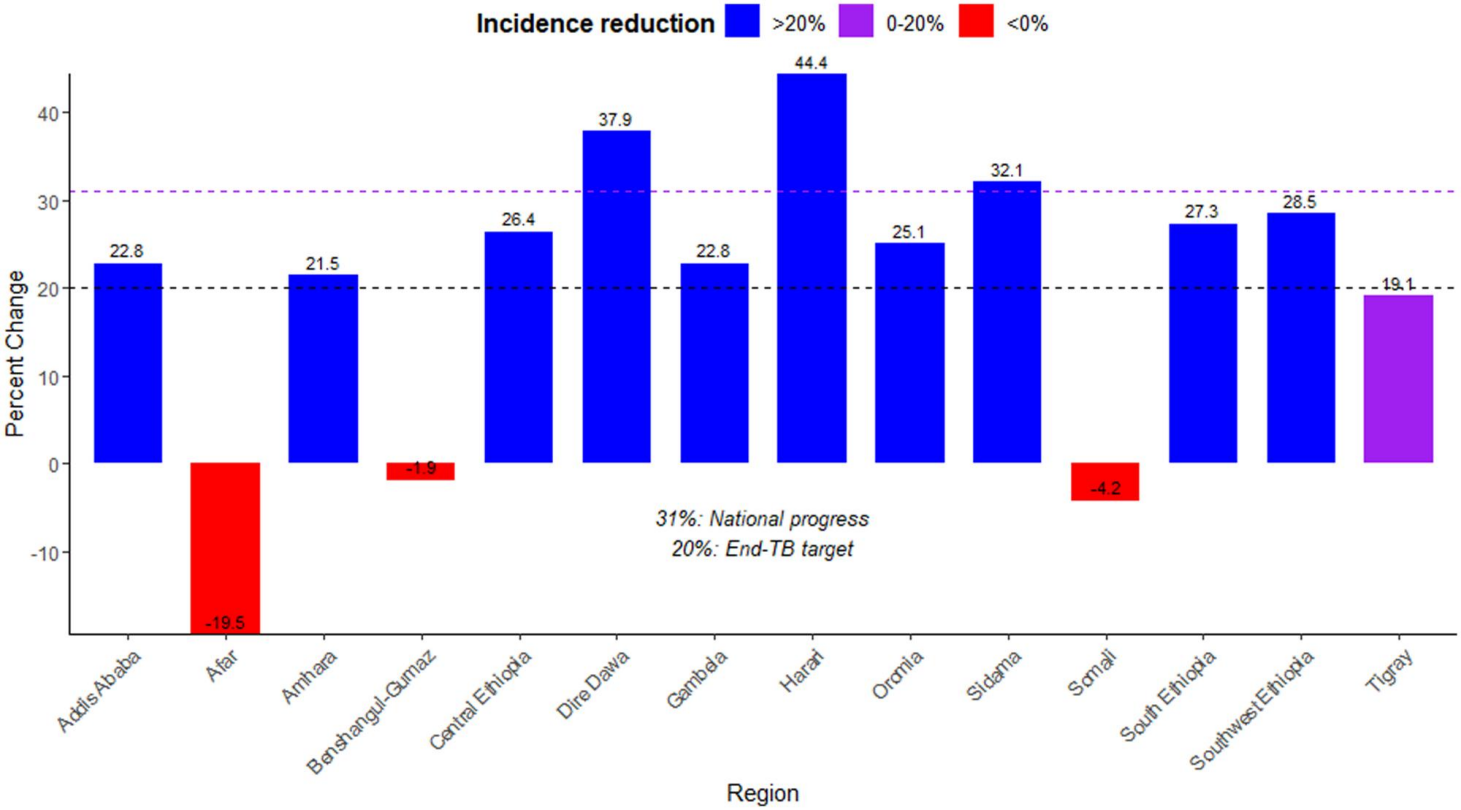
Regional-level average percentage change in incidence of tuberculosis between 2015 and 2020 in Ethiopia. The horizontal dashed line at 20 and 31 represents the WHO’s End TB incidence reduction target and the national average reduction in incidence, respectively. Negative values indicate an increase in tuberculosis incidence, while positive values indicate a decrease in incidence in 2020 compared with 2015.

Regional estimates of progress toward achieving the WHO’s End TB targets revealed that all regions except Dire Dawa, Harari, and Sidama showed incidence reduction below the national average, which was estimated to be 31%. Ten out of 14 regions achieved a reduction of >20% in TB incidence between 2015 and 2020. Among these, Harari exhibited the highest reduction, at 44%. Conversely, the Afar, Somali, and Benishangul-Gumuz regions experienced an increase in incidence during the same period (Fig. 1).

### Zone-level progress

**Figure 2.**
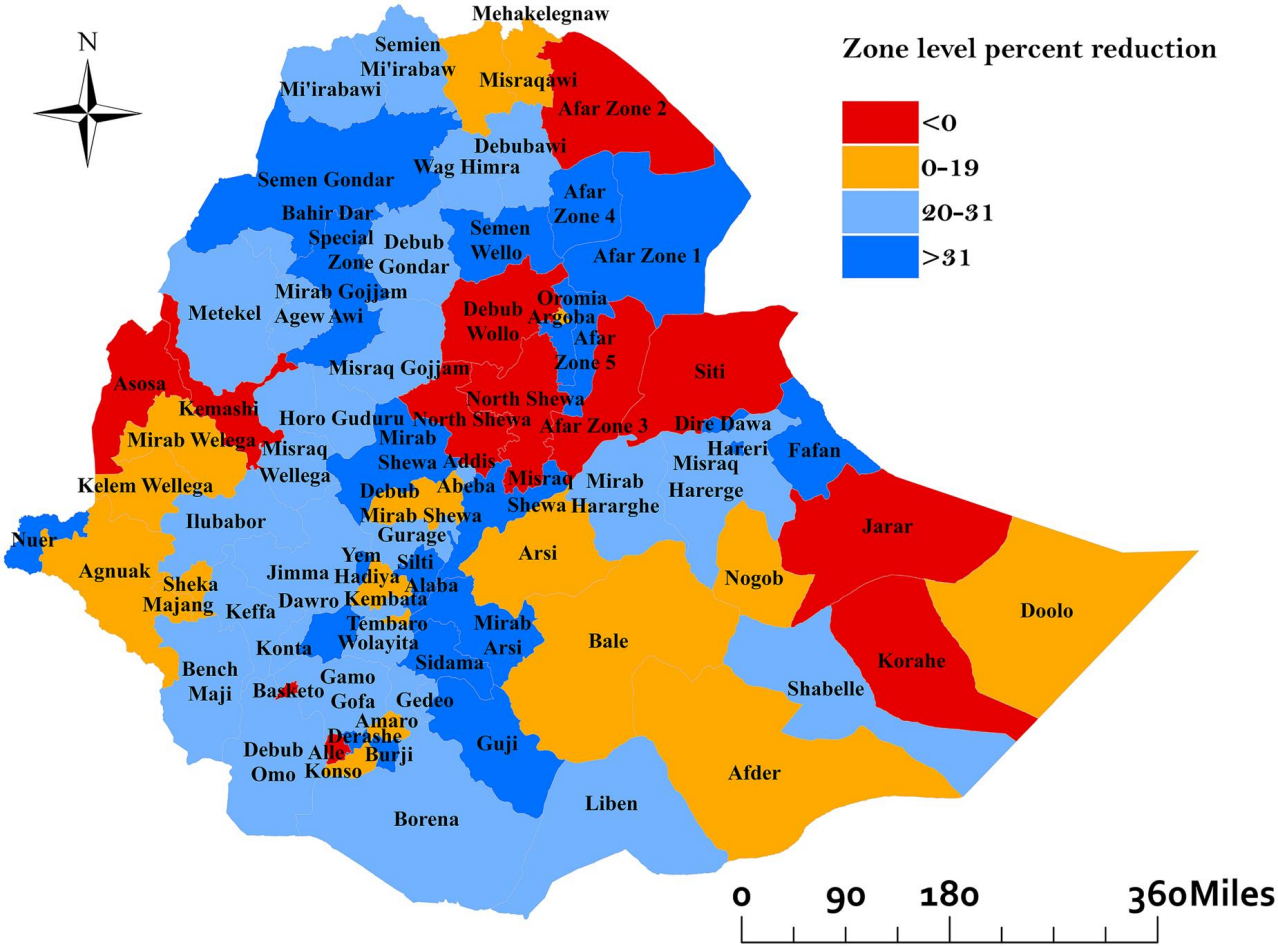
Zone-level average percentage reduction in incidence of tuberculosis between 2015 and 2020 in Ethiopia. Zones in the central and southern regions, particularly in Oromia and Southern Ethiopia, achieved the highest reductions (>31%). In contrast, zones in Afar, Somali, and parts of the western regions saw either minimal reductions or increases in tuberculosis incidence(<20%).

Of the 78 zones across the country, 11 zones (14.1%) experienced an increase in TB incidence between 2015 and 2020. Notably, the zones with the greatest increases were Zone 3 in the Afar region with a dramatic rise of 188.4%, Basketo Zone in South Ethiopia with an increase of 150%, and Siti Zone in the Central Ethiopia region with a rise of 78.3%. Conversely, 67 zones (85.9%) exhibited a reduction in TB incidence between 2015 and 2020. Among these, 50 zones (64.1%) met the WHO incidence reduction target. Furthermore, only 23 zones demonstrated a percentage reduction in TB incidence exceeding the national average of 31%. The zones with the most substantial reductions were Bahirdar (83.6%), Yem (82.7%), and Derashe (78.4%) (Fig. 2). The percentage change in incidence for all zones and regions in Ethiopia can be found in Supplementary Table S2.

### District-level progress

In our analysis, the incidence of TB either increased or showed no change in 136 out of the 641 districts, while it decreased in 508 districts, representing 79.3% of the total districts. However, only 395 (61.6) districts, mostly in the Oromia, Amhara, and South Ethiopia regions, achieved the WHO’s End TB target. Notably, districts such as Gonje in the Amhara region, Alamata in the Tigray region, and Gerar Jarso in the Oromia region demonstrated the highest reductions in incidence, with reductions of 98%, 93%, and 92.6%, respectively, between 2015 and 2020 (Supplementary Figure S1).

### Spatial analysis of TB incidence changes

**Figure 3.**
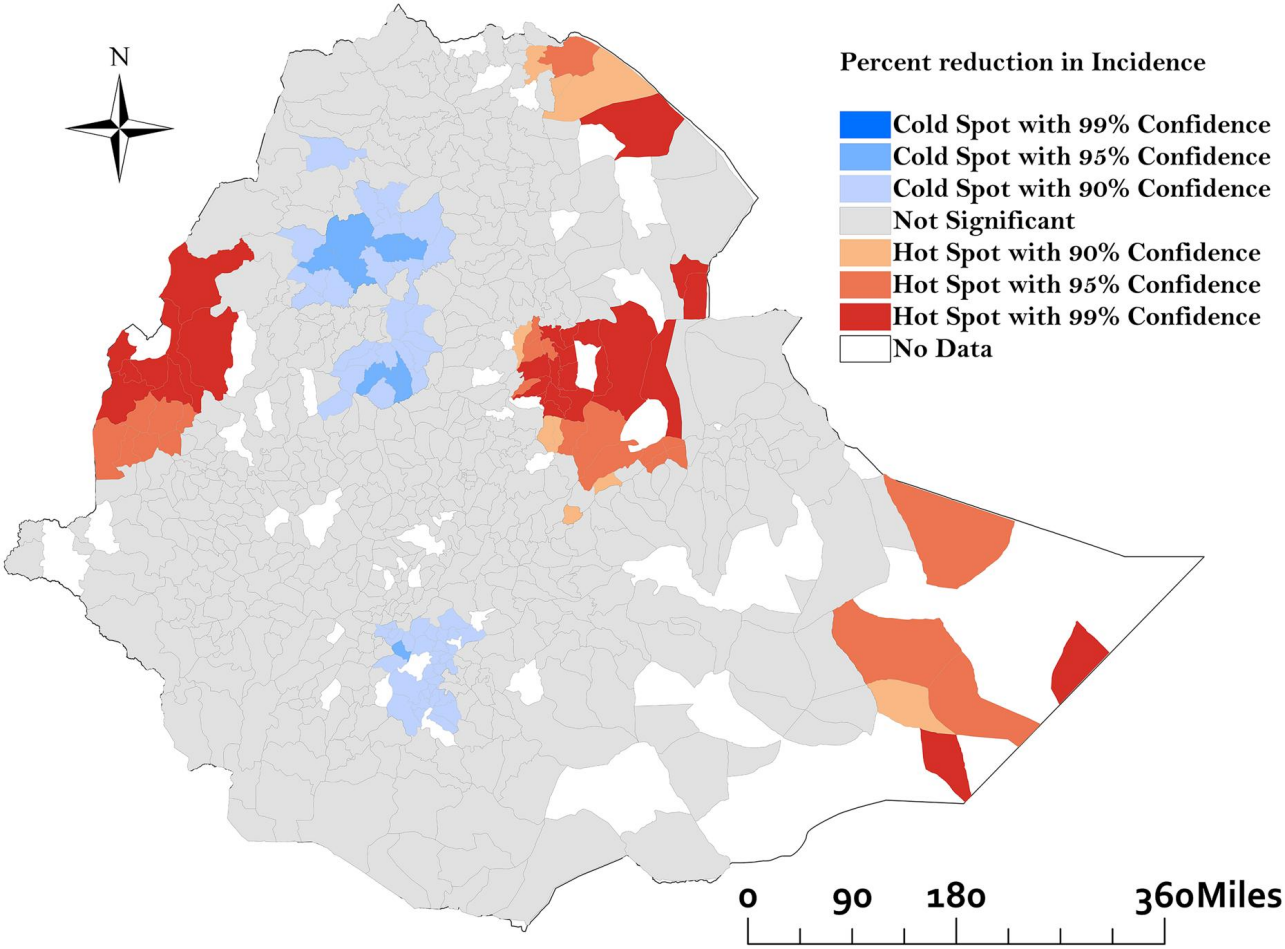
Spatial clustering of percentage reduction in tuberculosis incidence between 2015 and 2020 in Ethiopia based on the Getis-Ord statistics.

**Figure 4.**
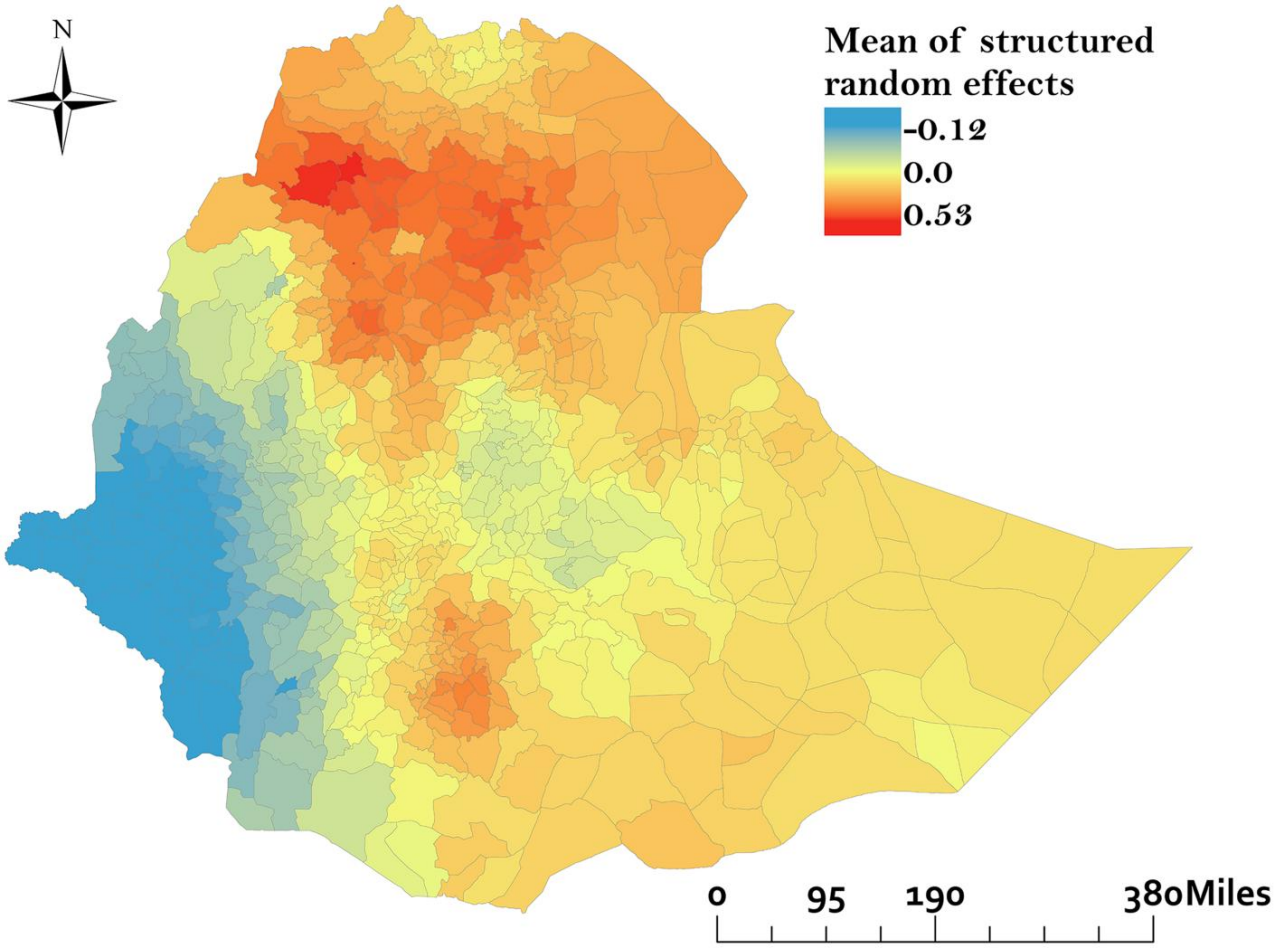
Posterior mean of spatially structured random effects for percentage reduction in tuberculosis incidence in Ethiopia.

The Global Moran’s I analysis revealed a positive spatial autocorrelation (Moran’s I = 0.02, *P* = .01), indicating that the decreased percentage changes in TB incidence between 2015 and 2020 were spatially clustered. Figure 3 shows the geographical distribution of hot and cold spots in TB incidence reduction across Ethiopia. Hot spots, highlight areas in which the TB incidence either increased or did not decrease as expected, with the darkest red indicating the highest confidence. These hot spots are primarily located in the north-eastern and south-eastern regions that are particularly prominent along the international borders with Eritrea and Djibouti. Cold spots, indicate areas with substantial reductions in TB incidence. These cold spots are mainly found in the South Ethiopia region, Amhara region, and seven districts in the Oromia region. Grey areas represent regions with no substantial change in TB incidence (Figure 3). The map of the posterior means of the spatially structured random effects demonstrates evidence of spatial clustering in TB incidence reduction after accounting for the model covariates. Many districts in the northern part of the country show smaller-than-expected reductions in TB incidence based on the included factors, while several districts in the western part of the country exhibit higher-than-expected incidence reductions (Figure 4).

### Factors associated with percentage reduction in TB incidence

Table 1 presents the results of a Bayesian multivariable linear regression model identifying factors associated with the percentage reduction in TB incidence in Ethiopia. The findings indicate that a 1% increase in the proportion of individuals with good knowledge about TB is associated with a 4.3% increase in TB incidence reduction [*β*: 4.3%; 95% credible interval (CrI): 1.6, 6.9]. Similarly, a 1-unit increase in the TB-specific service readiness index is associated with a 3.1% increase in TB incidence reduction (*β*: 3.1; 95% CrI: 0.9, 6.1). Additionally, a 1-km increase in the distance of a district from the international border is associated with a 2.3% increase in incidence reduction (*β*: 2.3%; 95% CrI: 0.02, 5.0) (Table 1). A table showing Bayesian logistic regression identifying factors associated with achieving the WHO’s TB incidence reduction targets (>20%) in Ethiopia is also included in Supplementary Table S3.

**Table 1.** Bayesian linear regression model for ecological-level factors associated with percentage reduction in incidence of tuberculosis in Ethiopia.

Variables	β (95% credible interval)
Good knowledge (%)	**4.3 (1.6, 6.9)**
TB services readiness index	**3.1 (0.9, 6.1)**
Precipitation (mm)	–0.08 (–3.0, 2.9)
Temperature (°C)	–1.4 (–4.4, 1.7)
Alcohol consumers (%)	1.4 (–0.8, 4.1)
Increased distance from the border (km)	**2.3 (0.02, 5.0)**

Bold: Statistically significant factors.

## Discussion

This study used advanced geospatial analysis to present the first local-level assessment of progress toward achieving the WHO’s End TB target for incidence reduction across Ethiopia between 2015 and 2020. Our findings reveal substantial spatial variations in TB incidence reduction at regional and local levels, associated with health service readiness, knowledge about TB, and distance of districts from international borders.

The observed spatial clustering of low reductions in TB incidence highlights that, while country-level estimates are valuable for international comparisons, they may obscure local geographic variations in progress towards achieving the WHO’s End TB targets. This suggests that TB remains poorly controlled in certain districts despite national achievement. Without the implementation of effective interventions, these hotspot districts could persist as sources of TB transmission, posing operational challenges to meeting the End TB target by 2035. Hence, it is crucial to design targeted interventions to address TB in these hotspot areas, focusing on strengthening cross-border collaboration, improving healthcare infrastructure, addressing socio-economic determinants, and enhancing surveillance and reporting mechanisms.

Our study has identified clusters of low TB incidence reduction in the Afar, Benishangul-Gumuz, and Somali regions, especially along international borders. This aligned with our findings that districts near international borders have decreased TB incidence reduction. These regions are characterized by the cross-border movement of people, including refugees, migrants, and nomadic populations, which could lead to the spread of TB across borders [35]. Border regions might experience political instability or conflict, which can disrupt health services, reduce TB control efforts, and lead to population displacement, further complicating TB prevention and treatment [36]. In addition, health facilities in these areas may be under-resourced, facing shortages of medical supplies, trained healthcare workers, and TB diagnostic tools, which can hinder effective TB control efforts [37]. Cultural barriers may have also contributed to poor TB control. For example, in the Somali region, traditional beliefs and self-treatment practices delay the biomedical diagnosis of TB [38], leading to ongoing transmission and poor incidence reduction. The regions have also been identified as areas of co-occurrence of high TB burden and low Bacille Calmette-Guérin (BCG) vaccine coverage, which could contribute to poor progress towards achieving the WHO’s End TB target [39]. Spatial clustering of TB incidence reduction was also observed after accounting for model covariates. Districts in the northern regions showed smaller-than-expected reductions, suggesting unmeasured barriers such as health system weaknesses or social determinants. In contrast, western districts demonstrated higher-than-expected reductions, potentially reflecting additional facilitators such as effective local interventions.

Our study found that districts with a higher proportion of people who are well-informed about TB are more likely to show increased TB incidence reduction. A well-informed population is more likely to engage in early health-seeking behaviours [40], adhere to treatment [41], and participate in TB control programmes, all of which enhance case detection and reduce transmission [42]. Additionally, increased awareness reduces stigma [43], encouraging timely medical intervention. Knowledgeable communities are also more likely to adopt preventive measures and support public health initiatives, creating an environment conducive to effective TB control and contributing to the achievement of incidence reduction goals. Hence, expanding educational programmes that are focused on TB awareness, particularly in the identified hotspot areas, could enhance public engagement in TB control measures, thereby improving the likelihood of meeting global TB reduction goals.

Our study has also found that districts with better preparedness and resources for TB service are more likely to have high TB incidence reduction. This is consistent with our finding from a global study that assessed the health system factors affecting progress toward achieving End TB targets [15]. Districts with strong health service readiness are likely to have advanced diagnostic capabilities, ensuring early and accurate TB detection, as well as improved access to essential medications and trained healthcare providers, leading to better treatment outcomes [44–46]. Additionally, these districts are better equipped to implement and sustain TB control programmes, provide preventive services, and integrate TB interventions within the broader health system [45]. Together, these factors create an environment that is conducive to effective TB control and contribute to achieving incidence reduction goals.

This study has some limitations. This study uses TB notifications as a proxy for TB incidence, which introduces certain limitations that should be acknowledged. While the stability of diagnostic practices and case detection efforts in Ethiopia during the study period (2015–20) supports the comparability of notifications over time, notifications may not fully capture the true incidence of TB. Decreasing notifications can reflect not only reductions in TB burden, but also potential declines in case detection or reporting. The lack of annual data for most independent variables made it impossible to conduct time-series analyses for each year. This limitation restricted our ability to explore year-to-year trends and understand the temporal dynamics of both TB incidence and its potential determinants, preventing us from capturing annual fluctuations, abrupt changes, or gradual trends that may have occurred between 2015 and 2020. Additionally, socio-economic factors, such as household quality, crowding, and income, were not included in our analysis due to the unavailability of district-level data, which further limited our ability to assess these important determinants of TB incidence reduction.

## Conclusions

Our study provides evidence of substantial spatial disparities in reducing TB incidence towards achieving End TB targets in Ethiopia. Regions along international borders such as Afar, Benishangul-Gumuz, and Somali are particularly lagging behind achieving the targets. These areas should be prioritized for targeted interventions, including improving TB service readiness and expanding TB awareness programmes.

## Ethics approval

Ethics clearance was obtained from Curtin University human research ethics office with approval number HRE2023-0250.

## Supplementary Material

dyaf157_Supplementary_Data

## Data Availability

The data underlying this article will be shared on reasonable request to the corresponding author.
