# Supplementary material for: Local progress towards achieving the End TB targets in Ethiopia: a geospatial analysis
Source: Int J Epidemiol. 2025 Sep 4;54(5):dyaf157. doi: 10.1093/ije/dyaf157 (PMC12410925; doi:10.1093/ije/dyaf157)
Supplement: dyaf157_Supplementary_Data [file dyaf157_supplementary_data.zip › ije-2024-12-2024-File008.docx]

**Supplementary file 2: Modelling details**

***Model Definition***

The Bayesian spatial linear regression model is defined as follows:

*Yi=α+βXi+Ui+Vi,i=1,2,…,n*

where:

- *Yi​:* Percent reduction in TB incidence for district i,
- *α:* Intercept term,
- *βXi​:* Fixed effects for covariates Xi,
- *Ui:* Spatially structured random effects,
- *Vi:* Unstructured random effects,
- *n:* Number of districts.

***Spatially Structured Random Effects (Ui​)***

The spatially structured random effects Ui are modeled using a Conditional Autoregressive (CAR) prior:


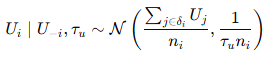


Where

- *U_-i_​:* Random effects for all districts except *i*,
- *δi​:* Set of neighboring districts for district *i*,
- *ni:* Number of neighbors for district *i*,
- *τu​:* Precision parameter for the spatially structured random effects.
- The joint distribution for *U* can be expressed as:


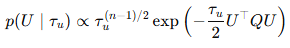


where *Q* is the precision matrix defined by the neighborhood structure:


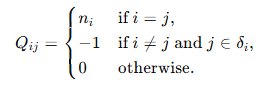


***Unstructured Random Effects (Vi)***

The unstructured random effects *Vi* are modeled as independent Gaussian random variables:


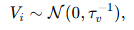


where *τv​* is the precision parameter for the unstructured random effects.

- **Intercept (α)**: Uniform prior (*U*(−∞,∞)),
- **Fixed effects (βj​)**: Normal prior (*βj*∼*N*(0,10^4^))
- **Precision parameters (*τu,τv*​)**: Gamma prior (*τ*∼Gamma(0.001,0.001))

**Conditional Autoregressive (CAR) Model**

A CAR model assumes that the value of a spatial random effect *Ui​* for a specific location iii depends on the values of the random effects in its neighboring locations. This dependency captures spatial autocorrelation, where locations that are close geographically tend to have similar values for the modeled random effects(1)

***Neighborhood Definition***

The neighbourhood structure is defined using an adjacency matrix *W*, where:


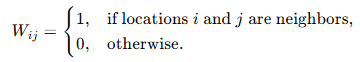


***CAR Model Specification***

The spatially structured random effects *U=(U1,U2,…,Un)​* are assumed to follow a joint multivariate normal distribution:


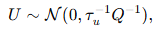


where:

- *τu*​: Precision parameter controlling the overall variability of the spatial random effects,
- *Q*: Precision matrix derived from the spatial weights matrix which is defined as:


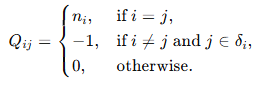


Here:

- *ni*: Number of neighbors for location *i*,
- *δi​:* Set of neighboring locations for *i*.

The structure of *Q* ensures that *U* exhibits spatial smoothing, where random effects at neighboring locations are more similar than at distant ones.

***Conditional Distribution***

The CAR model specifies the conditional distribution of *Ui* given all other *U_−I_* as:


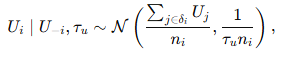


where:

- The mean is the average of the neighboring random effects,
- The variance depends inversely on *ni* (number of neighbors) and *τu* (precision parameter).

***Implementation in R***

The CAR model is implemented in INLA by specifying the spatial structure through the adjacency matrix *W*. INLA uses this information to construct the precision matrix *Q* and estimate the spatially structured random effects *U*.

##Load District shape file

map_ETH <- rgdal::readOGR("INPUT/ethiopia_districts/ethiopia_districts.shp")

# Load data

Deta_model <- read.csv("INPUT/Full_TB.csv")

DF <- data.frame(Deta_model)

#Merage data with shape file

map_DF <- merge(map_ETH, DF, by.x = "GID_3", by.y = "GID_3", all.x = TRUE )

##neigborhood structure

map_DF$idareau <- 1:nrow(map_DF)

map_DF$idareav <- 1:nrow(map_DF)

# Write the neighborhood structure to a file for INLA

nb2INLA("map.adj", map.nb)

# Read the adjacency file into INLA

g <- inla.read.graph(filename = "map.adj")

# Get the number of polygons (rows) in the neighborhood list

num_polygons <- length(map.nb)

print(num_polygons) # Display the number of polygons

# Fixed effects only

m1 <- inla(incidence_chage ~ 1 + scale(knowelage) + scale(wealth) + scale(temprature) + scale(service_readiness) +scale(tb_readiness) + scale(distance_to_border_km),

data = as.data.frame(map_DF), family = "gaussian",

control.predictor = list(compute = TRUE),

control.compute = list(dic = TRUE, waic = TRUE))

summary(m1)

# non-spatial (Spatial unstructured random effect)

m2 <- inla(incidence_chage~ 1 + scale(knowelage) + scale(wealth) + scale(temprature) + scale(service_readiness) +scale(tb_readiness) + scale(distance_to_border_km) + f(idareau, model = "iid"),

data = as.data.frame(map_DF), family = "gaussian",

control.predictor = list(compute = TRUE),

control.compute = list(dic = TRUE, waic = TRUE))

summary(m2)

# spatial- model (Spatial structured model)

m3 <- inla(incidence_chage~ 1 + scale(knowelage) + scale(rain) + scale(alcohol) + scale(temprature) + scale(tb_readiness) + scale(distance_to_border_km) +

f(idareav, model = "besag", graph = g),

data = as.data.frame(map_DF), family = "gaussian",

control.predictor = list(compute = TRUE),

control.compute = list(dic = TRUE, waic = TRUE))

summary(m3)

# Both spatially structured and unstructured model

formula <- incidence_chage ~ scale(Knowelage) + scale(Rain) + scale(Temprature) + scale(TB_readiness) + scale(Log) +

f(idareau, model = "besag", graph = g, scale.model = TRUE) +

f(idareav, model="iid")

# Run

m4 <- inla(formula, family = "gaussian", data = map_DF@data,

control.predictor = list(compute = TRUE),

control.compute = list(dic = TRUE, waic = TRUE))

summary(m4)

**Reference**

1. Besag J. Spatial interaction and the statistical analysis of lattice systems. Journal of the Royal Statistical Society: Series B (Methodological). 1974;36(2):192-225.
