# Supplementary material for: Local progress towards achieving the End TB targets in Ethiopia: a geospatial analysis
Source: Int J Epidemiol. 2025 Sep 4;54(5):dyaf157. doi: 10.1093/ije/dyaf157 (PMC12410925; doi:10.1093/ije/dyaf157)
Supplement: dyaf157_Supplementary_Data [file dyaf157_supplementary_data.zip › ije-2024-12-2024-File007.docx]

**Table S1:** Data sources and definitions of the variables used in the analysis.

| **Variables** | **Data sources** | **Definitions** |
| --- | --- | --- |
| Climatic variables | | |
| Mean temperature | WorldClime | Annual mean environmental air temperature (°C) |
| Mean precipitation | WorldClime | Annual mean rainfall (mm) |
| Wind speed | WorldClime | Annual mean wind speed (m s-1) |
| Health literacy indicator variables | | |
| Good knowledge towards TB | EDHS 2011 | Total number of people with good knowledge towards TB divided by the total number of people who participated in the survey. |
| Good attitude towards TB | EDHS 2011 | Total number of people with good attitude towards TB divided by the total number of people who participated in the survey |
| Good media exposure | EDHS 2016 | Total number of people exposed to at least one of the media including newspaper, radio or television at least once a week divided by the total number of people participated in the survey |
| Better Educational status | EDHS 2016 | Total number of the population who had attended primary and above education divided by the total number of people who participated in the survey. |
| Demographic and behavioural variables | | |
| High wealth index | EDHS 2016 | Total number of people with high wealth index (rich and richest) divided by the total number of people who participated in the survey. |
| Number of household members | EDHS 2016 | Average number of people living in a house |
| Cigarette smoking | EPHI STEPS | Total number of people currently smoke cigarettes divided by the total number of people participating in the survey |
| Alcohol drinking | EDHS 2016 | Total number of people drinking alcohol in the month prior to the survey divided by the total number of people participating in the survey |
| Chronic diseases | | |
| HTN | EPHI STEPS | Total number of people with HTN divided by the total number of survey participants |
| DM | EPHI STEPS | Total number of people with DM divided by the total number of survey participants |
| Service availability and readiness | | |
| General service readiness  and availability | EPHI SARA | Availability of equipment and supplies (i e, basic amenities,  equipment, standard precautions, diagnostic capacity, essential medicines) necessary to provide general health services |
| CRD readiness index | EPHI SARA | Availability of specific services for chronic respiratory disease (CRD) diagnosis, management and follow-up |
| TB readiness index | EPHI SARA | Availability of specific services for tuberculosis diagnosis, management and follow-up |
| Access to health facility | MAP | Walking travel times in minutes to the nearest health facility |

*EDHS, Ethiopia Demographic and Health Survey; EPHI, Ethiopia Public Health Institute; DM, Diabetes Mellitus; HTN, Hypertension; MAP, Malaria Atlas Project; SARA, Service Availability and Readiness Assessment*

**Table S2:** The percentage change in incidence of tuberculosis in Ethiopia at country, region, and zone levels, between 2015 and 2020.

|  | **Percent change in incidence of TB** |
| --- | --- |
| **Ethiopia** | 31.3 |
| **Addis Ababa** | 22.8 |
| **Afar** | -19.5 |
| Zone 01 | 36.6 |
| Zone 02 | -71.8 |
| Zone 03 | -188.4 |
| Zone 04 | 35.9 |
| Zone 05 | 34.8 |
| **Amhara** | 21.5 |
| South Gondar | 22.9 |
| North Wello | 32.1 |
| Oromiya Special zone | 37.7 |
| East Gojjam | 25.5 |
| North Gondar | 46.6 |
| South Wello | -2.9 |
| North Shewa | -4.8 |
| West Gojjam | 45.7 |
| Waghemira | 20.7 |
| Awi | 24.3 |
| Bahir Dar Special | 83.6 |
| Argoba Special | 5 |
| **Beneshangul Gumaz** | -1.9 |
| Metekel | 29.2 |
| Kamashi | -12.9 |
| Assosa | -27.1 |
| **Dire Dawa** | 37.9 |
| **Gambela** | 22.8 |
| Agnewak | 13.5 |
| Mejenger | 1.13 |
| Etang Special |  |
| Nuwer | 36.9 |
| **Harer** | 44.4 |
| **Oromiya** | 25.1 |
| Jimma | 28.4 |
| Arsi | 15.5 |
| East Wellega | 22.9 |
| Illu Aba Bora | 21.2 |
| West Wellega | 10.5 |
| West Shewa | 40.9 |
| East Shewa | 31.4 |
| North Shewa | -4.8 |
| West Hararge | 28.3 |
| East Hararge | 22.9 |
| Guji | 36.9 |
| Adama Special | 0.6 |
| Bale | 14.1 |
| West Arsi | 52.4 |
| Kelem Wellega | 11.5 |
| Horo Gudru Wellega | 29.3 |
| Borena | 24.6 |
| South West Shewa | 0.9 |
| **Central Ethiopia** | 26.4 |
| Gurage | 27.6 |
| Hadiya | 12.8 |
| Kembata Tembaro | 25.8 |
| Yem Special | 82.7 |
| Siliti | 33.5 |
| Alaba | 74.1 |
| **South-West Ethiopia** | 28.5 |
| Keffa | 27.4 |
| Sheka | 3.1 |
| Bench Maji | 23.4 |
| Amaro Special | 17.8 |
| Dawro | 42.1 |
| Konta | 27.0 |
| Sidama | 32.1 |
| South Ethiopia | 27.3 |
| Gedeo | 30.6 |
| Gamo Gofa | 30.5 |
| Wolayita | 26.6 |
| South Omo | 24.1 |
| Burji Special | 44.7 |
| Konso Special | 8.1 |
| Derashe Lyiu | 78.5 |
| Basketo | -150 |
| **Somali** | -4.2 |
| Afder | 12.01 |
| Korahe | -63.3 |
| Jarar | -60.2 |
| Doolo | 3.3 |
| Shabelle | 26.7 |
| Nogob | 3.2 |
| Fafan | 58.2 |
| Siti | -78.3 |
| Liben | 20.0 |
| **Tigray** | 19.1 |
| North Western Tigray | 24.0 |
| Eastern Tigray | 16.1 |
| Central Tigray | 15.4 |
| South Tigray | 21.5 |
| Western Tigray | 21.7 |

1. b.


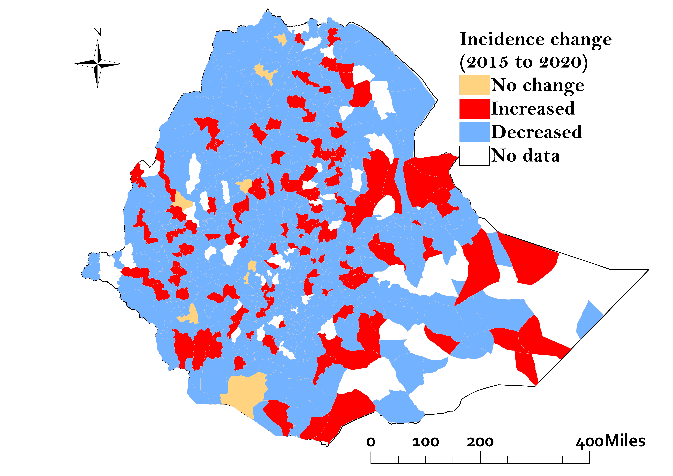

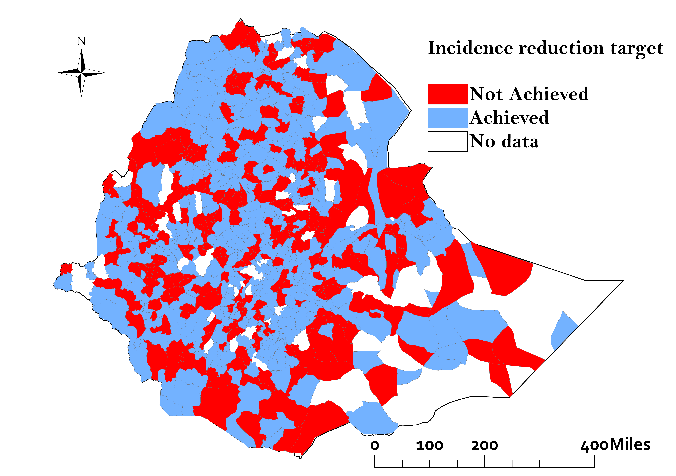


**Figure S1:** Change in tuberculosis incidence (a) Progress toward World Health Organisation tuberculosis Incidence Reduction Target (b) between 2015 and 2020 at district level in Ethiopia.

*Districts with a percent change in incidence of tuberculosis ≥ 20% were categorized as having achieved the target*

**Table S3:** Bayesian spatial logistic regression model for factors associated with the achievement of the World Health Organization tuberculosis incidence reduction target in Ethiopia.

| *Variables* | *OR (95% Credible Interval)* |
| --- | --- |
| Good knowledge | **4.16 (2.90, 5.98)** |
| High wealth | 0.75 (0.50, 1.12) |
| Smoking | 1.12 (0.77, 1.64) |
| General service readiness | **8.33 (5.26, 13.33)** |
| TB services readiness | **3.74 (2.59, 5.37)** |
| Increased distance from border | **4.18 (2.46, 7.02)** |
| Temperature | 1.32 (0.79, 2.19) |
| Precipitation | **1.63 (1.11, 2.39)** |

*Bold: Statistically significant variables*
